# Encapsulation of Rosehip (*Rosa canina* L.) Seed Polyphenols in Polysaccharide‐Based Carriers: Process Optimization, Characterization, and In Vitro Release

**DOI:** 10.1002/fsn3.71376

**Published:** 2025-12-19

**Authors:** İrem Toprakçı, Ebru Kurtulbaş, Mehmet Torun, Selin Şahin, Seid Reza Falsafi

**Affiliations:** ^1^ Chemical Engineering Department, Faculty of Engineering Istanbul University‐Cerrahpasa Istanbul Türkiye; ^2^ Food Engineering Department, Faculty of Engineering Akdeniz University Antalya Türkiye; ^3^ Agricultural Engineering Research Department, Food Science and Technology Division Safiabad Agricultural and Natural Resources Research and Education Center, (AREEO) Dezful Iran

**Keywords:** chemometric approach, natural antioxidants, polyphenols, spray‐drying

## Abstract

This study investigated to enhance the value of rosehip ( *Rosa canina* L.) seed as a source of phenolic antioxidants for application in model food systems (sunflower and corn oils). Encapsulation formulations were developed using a mixture design with two coating materials (Arabic gum and maltodextrin) and one process factor (inlet temperature) to optimize encapsulation efficiency (EE) with respect to total polyphenols, microencapsulation yield (EY), and antioxidant activity. The model was highly significant (*p* < 0.0001) with no lack of fit (*p* > 0.05), while the optimum conditions (Arabic gum at 160°C) were validated with < 2% deviation. The developed microparticles exhibited 66.08%–84.49% EE, 53.57%–73.67% EY, and 3.72–4.21 mg‐TEAC/g‐DM antioxidant activity. SEM confirmed the morphological uniformity, whereas chemometric analysis effectively differentiated the formulations. In vitro digestion showed polyphenol release was higher in the intestinal phase (68.47%) than in the gastric phase (65.44%). Incorporation of the encapsulated powders enhanced the oxidative stability of sunflower and corn oils by 32% and 60%. These results demonstrate that spray‐drying encapsulation is an efficient and sustainable strategy to stabilize rosehip seed polyphenols and improve their applicability in functional food formulations.

## Graphical abstract

Rosehip seed polyphenols were encapsulated by optimized spray‐drying using polysaccharide carriers. The microparticles showed strong antioxidant properties and higher release in the intestinal phase. Their incorporation improved the oxidative stability of sunflower and corn oils.

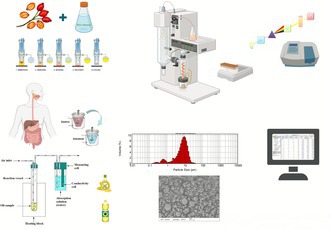

## 1 Introduction

Polyphenols are important bioactive compounds naturally found in plant materials, and their regular consumption has been associated with reduced risk of chronic diseases such as cancer, osteoporosis, cardiovascular disorders, and diabetes (Li et al. 2021). These compounds exhibit multiple biological activities, including antioxidant, anti‐inflammatory, antifungal, antibacterial, and antiviral properties (Sharma 2014). In particular, their antioxidant activity is critical in protecting DNA from oxidative stress and preventing structural deterioration (Rathod et al. 2023).

Despite these benefits, the direct application of polyphenols in food systems is limited because of their generally low concentrations, poor water solubility, and high sensitivity to environmental factors such as light, oxygen, heat, pH, and moisture during processing and storage (Albuquerque et al. 2021). Moreover, their stability, bioactivity, and sensory properties are influenced by interactions with food components (Delfanian and Sahari 2020), while their bioavailability can be further reduced by factors such as the food matrix, gastrointestinal digestion, metabolism, and tissue distribution. Therefore, strategies are required to improve the stability and bioavailability of polyphenols and ensure their effective delivery in functional food applications. Encapsulation has emerged as a promising approach to overcome these limitations. By coating or entrapping bioactive compounds within carrier materials, encapsulation can protect polyphenols from environmental stresses, enhance their stability, mask undesirable flavors, and improve their controlled release (Grgić et al. 2020; Macías‐Cortés et al. 2020; Garavand et al. 2021). Among various methods, spray‐drying is widely used in the food industry because it is cost‐effective, scalable, and suitable for a wide range of wall materials (Zuidam and Nedović 2010). Its advantages include simple operation, continuous production, efficient retention of active substances, and good product stability (Jayaprakash et al. 2023; Piñón‐Balderrama et al. 2020).

Rosehip ( *Rosa canina* L.) seed was selected in this study as a polyphenol source. The seed, a byproduct that constitutes nearly 30% of the fruit, is often discarded as waste (Salgın et al. 2016). Most research has focused on recovering oil from rosehip seeds (Dąbrowska et al. 2019; Milić et al. 2020; Salgın et al. 2016), whereas limited studies have investigated their polyphenol‐rich extracts. Yet, Ilyasoğlu (2014) demonstrated that rosehip seed extract contains a significant amount of polyphenols (2554 μg/g) with notable antioxidant activity. This highlights the potential of rosehip seed extract as a valuable, underutilized source of bioactive compounds.

Therefore, in the present study, rosehip seed extract was encapsulated using Arabic gum, maltodextrin, and their blend as wall materials via spray‐drying. Process optimization was carried out to maximize encapsulation efficiency and preserve antioxidant activity, while morphological, physicochemical, and release properties of the resulting microparticles were characterized. Furthermore, the in vitro release behavior of the encapsulated polyphenols was evaluated to assess their potential bioavailability. This work represents the first attempt to valorize rosehip seed waste as a source of polar antioxidants stabilized by spray‐drying, providing new opportunities for its use in functional food applications.

## 2 Materials and Methods

### 2.1 Materials

Rosehip ( *Rosa canina* L.) fruits were obtained from Çankırı in Türkiye (2024). The seeds were separated from the fresh samples and dried at ambient conditions. Additionally, samples of sunflower oil and corn oil were procured from a nearby store.

The chemical materials used in the tests and experiments came from Merck (Darmstat, Germany) and Sigma‐Aldrich (St. Louis, MO, USA). They were ethanol (≥ 99.8%), methanol (≥ 99.9%), phosphate buffer (pH 7.4), sodium carbonate (Na_2_CO_3_), sodium chloride (NaCl), hydrochloric acid (HCl), Folin–Ciocalteu reagent, gallic acid, DPPH, ABTS, trolox, maltodextrin, and Arabic gum.

### 2.2 Extraction of Bioactive Substance From Rosehip Seeds

A solvent autoextractor (VELP Scientifica, Usmate, Italy) was utilized for the extraction of active substances from dried samples. The process took 90 min to finish. The temperature was approximately 80°C. Cellulose thimbles (33 mm × 80 mm, Whatman, Maidstone, UK) were used to hold the plant samples (1 g). These were then kept in glass liquid cups. Ethanol solution (80%, v/v) was used to extract the bioactives from rosehip seeds.

### 2.3 Microparticle Production

The microencapsulation of the rosehip seed extract was conducted using laboratory‐scale apparatus (B‐290; Buchi, Switzerland). Maltodextrin, Arabic gum, and a combination of the two were chosen as wall materials. So, statistical experimental design was implemented using the mixture design approach. The proportions of maltodextrin and Arabic gum in the mixture (0%, 2.5%, 5%, 7.5%, and 10%; w/v) were independent factors in this process. Additionally, the drying air inlet temperature was also an independent factor (130°C, 145°C, 160°C, 175°C, and 190°C). In order to maintain a consistent discharge temperature, the flow rate of the solution was adjusted to 240–640 mL per hour. Outlet temperature (85°C ± 5°C), nozzle pressure (0.5 MPa), and aspiration rate (30 m^3^/h (80%)) remained constant during the process.

Encapsulation yield (EY) is a metric for assessing the performance of the spray‐drying. The calculation is performed as Equation (1):

(1) $$\mathrm{EY}\left(\%\right)=\frac{\text{Weight of collected powder}}{\text{Weight of solid feeding extract}}\times 100$$

### 2.4 Biochemical Evaluation

Encapsulation efficiency (EE) was evaluated based on total polyphenolic content (TPC) as outlined by Škerget et al. (2005). TPC values were determined under 760 nm. The surface phenolic content (SPC) of the products was then computed to express the EE results (Tolun et al. 2016). EE is determined by Equation (2):

(2) $$\mathrm{EE}\left(\%\right)=\frac{\mathrm{TPC}-\mathrm{SPC}}{\mathrm{TPC}}\times 100$$

Furthermore, the DPPH technique (517 nm) was used to assess the antioxidant activity of the microparticles (Tolun et al. 2016).

### 2.5 Characterization of the Microparticles

Following the production of microparticles under optimal conditions, the samples were subjected to a variety of physicochemical analyses [bulk density, tapped density, water activity, moisture content, solubility, and Carr index (CI)].

The mass/volume ratio was used to determine the bulk density (*ρ*_b_) of the samples. The 2 g sample was placed in the 10 mL measuring tape after it had been weighed. The tapped density (*ρ*_t_) of the samples was also determined by measuring the volume after hitting the cylinder‐shaped container with a 2 g sample 35 times on a hard surface by hand and pressing it down to get rid of any holes between the powders. It is given in kg/m^3^ for *ρ*_b_ and *ρ*_t_. The CI values of the powder samples were found using Equation (3) (Beristain et al. 2001):

(3) $$\mathrm{CI}=\frac{\rho_{t}-\rho_{b}}{\rho_{t}}\times 100$$

The particle's moisture content was ascertained gravimetrically using a fast moisture analyzer (Kern DBS 60‐3, Balingen, Germany). The data were computed as percentage dry matter (% DM). A water activity detector (AquaLab, 4TE, USA) was used to measure the microparticle's water activity value at room temperature.

A 0.5‐g sample was used for the solubility analysis of particle samples. 50 mL of water was incorporated and stirred. Following centrifugation (3000 *g* and 5 min), the residual phase was maintained at 70°C until a consistent weight was achieved. The solubility (%) was used to express the weight difference (Şahin‐Nadeem et al. 2013).

### 2.6 Particle Size Analysis

Mastersizer 2000 software (Malvern, Worcestershire, UK) was utilized to measure particle size using the light scattering approach. The microcapsules were dispersed in 2‐propanol (1%) in order to do the measurements. Equation (4) was used to determine the powder product's particle size, which was represented as the mean volumetric area size (*D*_[4,3]_):

(4) $$D_{\left[4,3\right]}=\frac{\sum n_{i}{d_{i}}^{4}}{\sum n_{i}{d_{i}}^{3}}$$

*n*_*i*_ represents the quantity of particles with diameter *d*_*i*_.

Span denotes the dispersion of particles within the heap, and is determined using Equation (5):

(5) $$\text{Span}=\frac{d_{90-}d_{10}}{d_{50}}$$

*d*_90_, *d*_10_, and *d*_50_ denote equivalent volumetric diameters corresponding to 90%, 10%, and 50% cumulative volume, respectively.

### 2.7 Scanning Electron Microscopy

The morphological measurement was conducted using a scanning electron microscope (SEM) (Carl Zeiss Leo 1430, Germany). Powder samples were fixed on aluminum stubs with double‐sided carbon adhesive tape and gently tapped to remove loose particles. Then, the samples were coated with a thin (~10 nm) conductive layer of gold and palladium using a sputter coater under a vacuum of approximately 0.1 mbar for 60 s to prevent electrostatic charging during analysis. Micrographs were captured at magnifications of 1.00K, 3.00K and 5.00K to assess particle shape, surface smoothness and agglomeration.

### 2.8 Mixture Design and Statistical Analysis

The statistical experimental design and optimization were conducted using mixture design, since it is more appropriate for systems that contain mixtures (Goos et al. 2016). The software used was Design‐Expert (12.0.1.0 version, StatEase Inc., USA). A and B represent the mixture components corresponding to Arabic gum ratio and maltodextrin, respectively, while the spray‐drying inlet temperature is another factor (C). Y shows the dependent variable (response) of the process. The optimization was performed to maximize EE, EY, and antioxidant activity of the powder samples. Y_EE_, Y_EY_, and Y_antioxidant activity_ denote the metrics for EE, EY, and antioxidant activity, respectively. While Table 1 presents the independent and dependent variables along with their coded levels. Additionally, the findings were statistically assessed using the Analysis of Variance (ANOVA) test via the Design‐Expert software. Model fitting and the effects of the parameters were analyzed by ANOVA.

**TABLE 1:** Process parameters for microencapsulation of rosehip seed extract by spray‐drying method.

Process parameters	Coded levels				
	−2	−1	0	1	2
A, Arabic gum (%, w/v)	0	2.5	5	7.5	10
B, Maltodextrin (%, w/v)	0	2.5	5	7.5	10
C, Temperature (°C)	130	145	160	175	190

Responses	Goal
Encapsulation efficiency (%)	Maximize
Encapsulation yield (%)	Maximize
Antioxidant activitiy (mg‐TEAC/g‐DM)	Maximize

### 2.9 Chemometric Analysis

Principal component analysis (PCA) is a statistical method to reduce the number of dimensions. It does this by breaking down a big set of variables into a smaller set of variables that are not related to each other. These variables are called principal components (Sharma and Kumar 2019). It is a classical technique that involves orthogonal transformation to create a new set of coordinates that are linearly uncorrelated. PCA is essential in various fields such as pattern recognition, image processing, and data compression due to its ability to reduce the number of variables while preserving the most critical information in the data (Arabaci and Laving 2019).

In this study, different encapsulation formulations were developed by means of mixture design and 19 different microparticles were prepared and evaluated by means of PCA. This technique allows insight into the characteristics of microcapsules with different compositions, as well as helping to resolve complex data correlations. XLSTAT (trial version) was used to perform the PCA method.

### 2.10 Polyphenol Release Test

In vitro testing is essential for obtaining preliminary information prior to the in vivo phase. TPC release experiments from polyphenol‐loaded microparticles were performed in synthetic gastric fluid (SGF) and synthetic intestinal fluid (SIF). The SGF had an acidic pH around 1.5, whereas the SIF had a more neutral pH of 7.4 (Zhang and Kosaraju 2007). Approximately 0.2 g of sample was added to 50 mL of SGF and SIF solutions, respectively. The samples were placed in a shaking water bath (Nüve, ST‐402) at 37°C to complete the release process. At consistent intervals, 1.5 mL of the sample was extracted and substituted with the corresponding solution.

### 2.11 Application of the Microparticles Into the Vegetable Oils

The application of microparticles as an ingredient enhanced the stability and functional characteristics of the vegetable oil samples (corn and sunflower oil). Following the enrichment of the oil samples, the samples were examined for oxidative stability and bioactive characteristics. The Folin method and the ABTS (2,2′‐azino‐bis‐(3‐ethylbenzothiazoline‐6‐sulfonic) acid) assay were used to determine the samples' TPC and antioxidant activity (bioactive characteristics), while the Rancimat method (an accelerated oxidation test) was employed to measure the oxidative stability.

Additionally, one‐way ANOVA was employed to discern the differences among groups utilizing Minitab Statistical Software 22 (Minitab Inc., State College, PA, USA). Tukey's test was employed for post hoc pairwise comparisons. Confidence intervals of 95% (*α* = 0.05) were computed to assess the accuracy of the mean differences.

#### 2.11.1 Accelerated Oxidation Test

The accelerated oxidation test was conducted using the Rancimat device (Metrohm, 892, Herisau, Switzerland) at a temperature of 140°C. The duration necessary for the identification of the secondary oxidation reaction products is referred to as the induction time (IT). This value provides an understanding of the stability of the oil‐containing items.

#### 2.11.2 Total Phenolic Content Test

We employed the Folin–Ciocalteu method to evaluate total phenolic content of the enriched and pure oil samples. After the products were combined with hexane using a digital homogenizer (IKA T25 Ultra Turrax, Germany), methanol was used to extract the phenolic components from the oil samples. After centrifugation, the methanolic extracts were reacted with the Folin–Ciocalteu reagent and Na_2_CO_3_ solution. Then, the absorbance was measured at 765 nm spectrophotometrically. Results were expressed as mg of gallic acid equivalents per g oil sample (mg‐GAE/g‐OS).

#### 2.11.3 Antioxidant Activity Test

Based on our initial testing, the ABTS assay produced more repeatable results than the DPPH assay. That's why the ABTS method was employed for the oil samples. Methanolic extracts of the oils were prepared as described above and reacted with ABTS radical solution. The absorbance decrease at 734 nm was recorded after 15 min. The antioxidant activity was expressed as mg of Trolox equivalents per g of sample (mg‐TEAC/g‐OS).

## 3 Results and Discussion

### 3.1 Spray‐Drying of Rosehip Seed Polyphenols in Arabic Gum/Maltodextrin

Table 2 presents the design matrix of the spray‐drying process of rosehip seeds polyphenols. Maltodextrin is a popular coating material in encapsulation systems due to its advantageous properties such as low viscosity, colorless appearance, high water solubility, and low sugar content (Akhavan Mahdavi et al. 2016). On the other hand, Arabic gum is another preferable coating agent owing to being colorless, highly soluble, biocompatible, optimum viscose (Sarabandi et al. 2019). Earlier works observed that the mixture of maltodextrin and Arabic gum yielded better than alone (Akhavan Mahdavi et al. 2016; Pratami et al. 2019; Sarabandi et al. 2019; Tan et al. 2015).

**TABLE 2:** Mixture design matrix for the microencapsulation of rosehip seed extract by spray‐drying method.^a^

Run	Arabic gum (%, w/v)	Maltodextrin (%, w/v)	Temperature (°C)	EE_TPC_ (%)	EY (%)	DPPH (mg‐TEAC/g‐DM)
1	2.5	7.5	145	73.49	61.95	3.81 ± 0.003
2	5	5	160	72.70	61.06	3.91 ± 0.005
3	2.5	7.5	130	79.83	62.71	3.72 ± 0.001
4	7.5	2.5	145	74.92	60.01	4.03 ± 0.002
5	5	5	190	77.52	53.57	3.76 ± 0.002
6	10	0	130	66.77	56.69	4.21 ± 0.004
7	0	10	160	75.95	62.27	3.88 ± 0.001
8	0	10	190	66.08	65.10	3.85 ± 0.002
9	2.5	7.5	175	72.05	59.58	3.85 ± 0.003
10	5	5	130	79.81	54.51	3.75 ± 0.003
11	7.5	2.5	175	77.43	60.74	4.03 ± 0.002
12	10	0	190	71.25	58.31	4.07 ± 0.004
13	10	0	160	84.49	63.66	4.18 ± 0.005
14	0	10	160	74.43	62.63	3.82 ± 0.006
15	10	0	160	83.81	63.99	4.18 ± 0.002
16	5	5	190	80.38	54.75	3.86 ± 0.006
17	5	5	160	73.39	61.53	3.94 ± 0.002
18	0	10	130	78.06	73.67	3.80 ± 0.003
19	5	5	160	74.92	60.83	3.93 ± 0.005

^a^ Data are given as the arithmetic mean of three replicates.

The EE in terms of TPC changed from 66.08% (run 8) to 84.49% (run 13). The highest inlet temperature and the pure maltodextrin gave the least yield. So, we can conclude that the coating material affects the process significantly. Šturm et al. also reported that Arabic gum alone showed the best performance for encapsulation of the propolis extract due to the glycoprotein fraction in the structure of Arabic gum compared to maltodextrin and the mixture of maltodextrin and Arabic gum (Šturm et al. 2019). EY of the spray‐drying process varied from 53.57% (run 5) to 73.67% (run 18) depending on the conditions (Table 2). Similarly, inlet temperature impacts the yield negatively. Antioxidant activity values were between 3.72 (run 3) and 4.21 mg‐TEAC/g‐DM (run 6) as seen in Table 2. Similar to EE_TPC_, the antioxidant activity produced the highest yield when Arabic gum was utilized alone. This characteristic can be explained by the hydrophilic gum's (Arabic gum) good film‐forming qualities, which prevent antioxidants from escaping the capsule shell (Lu et al. 2021). Arabic gum may offer superior morphology to preserve the bioactives in the capsule (Murugesan and Orsat 2011).

### 3.2 Mixture Design of Spray‐Drying Process of Rosehip Seed Polyphenols

Table 3 is the combined model fit summary table. The selected models (quadratic × quadratic) have non‐significant lack of fit. Furthermore, the chosen models are maximizing the adjusted *R* ^2^.

**TABLE 3:** Combined model fit summary.^a^

	Mixture order	Process order	Mixture *p* value	Process *p* value	Lac of fit *p* value	Adjusted *R* ^2^	Predicted *R* ^2^	
EE	M	M						
	M	L		0.5717	0.0016	−0.0385	−0.2736	
	M	Q		0.5245	0.0014	−0.0750	−0.4448	
	M	C		0.7666	0.0012	−0.1397	−0.5250	
	L	M	0.3968		0.0017	−0.0138	−0.3061	
	L	L	0.1242	0.1489	0.0021	0.1087	−1.0292	
	L	Q	0.0431	0.1006	0.0031	0.2778	−1.4704	
	L	C	0.1248	0.9630	0.0018	0.1523	−2.0090	
	Q	M	0.6471		0.0014	−0.0627	−0.5055	
	Q	L	0.7437	0.2778	0.0015	0.0174	−3.0109	
	Q	Q	< 0.0001	< 0.0001	0.6674	0.9448	0.8975	Suggested
	Q	C	< 0.0001^b^	0.7053^b^	0.5124	0.9367	0.0680	Aliased
	C	M	0.9547		0.0012	−0.1333	−0.6066	
	C	L	0.9295	0.4675	0.0009	−0.1459	−3.8742	
	C	Q	0.9855	< 0.0001	0.2938	0.9226		
	C	C	0.8941^b^	0.4467^b^	0.1859	0.9187		Aliased
EY	M	M						
	M	L		0.2284	< 0.0001	0.0303	−0.2879	
	M	Q		0.3568	< 0.0001	0.0245	−0.4543	
	M	C		0.8142	< 0.0001	−0.0365	−0.5154	
	L	M	0.1054		< 0.0001	0.0966	−0.0944	
	L	L	0.1543	0.2626	< 0.0001	0.1434	−0.4995	
	L	Q	0.0185	0.0282	< 0.0001	0.4292	−1.6408	
	L	C	0.0651	0.9802	< 0.0001	0.3279	−2.3137	
	Q	M	0.0159		< 0.0001	0.3402	0.1129	
	Q	L	0.0493	0.3089	< 0.0001	0.3780	−1.1726	
	Q	Q	< 0.0001	< 0.0001	0.3963	0.9880	0.9014	Suggested
	Q	C	< 0.0001^b^	0.9219^b^	0.2245	0.9853	0.5351	Aliased
	C	M	0.7659		< 0.0001	0.3005	0.0679	
	C	L	0.8423	0.4807	< 0.0001	0.2875	−1.5038	
	C	Q	0.1559	< 0.0001	0.7365	0.9915		
	C	C	0.1148^b^	0.6396^b^	0.5406	0.9905		Aliased
Antioxidant activity	M	M						
	M	L		0.8685	0.0012	−0.0571	−0.2923	
	M	Q		0.2349	0.0013	−0.0255	−0.3665	
	M	C		0.9435	0.0010	−0.0935	−0.4898	
	L	M	< 0.0001		0.0174	0.6501	0.5886	
	L	L	< 0.0001	0.3869	0.0162	0.6506	0.5162	
	L	Q	0.0003	0.2145	0.0181	0.6819	−0.2524	
	L	C	0.0025	0.9651	0.0106	0.6264	−0.5652	
	Q	M	0.0007		0.0773	0.8231	0.7846	
	Q	L	0.0026	0.2595	0.0838	0.8384	0.7178	
	Q	Q	< 0.0001	0.0004	0.9563	0.9639	0.9034	Suggested
	Q	C	0.0001^b^	0.9876^b^	0.8235	0.9550	−0.1768	Aliased
	C	M	0.2903		0.0769	0.8253	0.7843	
	C	L	0.6256	0.4543	0.0617	0.8247	0.6810	
	C	Q	0.7879	0.0068	0.9318	0.9552		
	C	C	0.6582^b^	0.9343^b^	0.7265	0.9478		Aliased

^a^ M: Mean; L: Linear; Q: Quadratic; C: Cubic.

^b^ The combined model is aliased.

Table 4 gives the analysis of variance test results for the responses. Equations ((6), (7), (8)) are the models derived by the Design‐Expert software.

(6) $$\begin{array}{c} Y_{\mathrm{EE}}=84.07A+74.98\;B-25.71\;\mathrm{AB}+2.28\;\mathrm{AC}-5.88\;\mathrm{BC}\\ \;+7.23\;\mathrm{ABC}-15.07\;AC^{2}-3.02\;BC^{2}+59.72\;\mathrm{AB}C^{2} \end{array}$$

(7) $$\begin{array}{c} Y_{\mathrm{EY}}=63.94\;A+62.37\;B-7.79\;\mathrm{AB}+0.8812\;\mathrm{AC}-4.45\;\mathrm{BC}\\ \;+5.28\;\mathrm{ABC}-6.45\;AC^{2}+7.12\;BC^{2}-27.64\;\mathrm{AB}C^{2} \end{array}$$

(8) $$\begin{array}{c} Y_{\text{antioxidant activity}}=4.19\;A+3.85\;B-0.3514\;\mathrm{AB}-0.0705\;\mathrm{AC}\\ \;+0.0256\;\mathrm{BC}+0.2005\;\mathrm{ABC}-0.0473AC^{2}\\ \;-0.0251\;BC^{2}-0.4329\;\mathrm{AB}C^{2} \end{array}$$

**TABLE 4:** Analysis of variance findings on encapsulation efficiency value of microcapsules.

	Source	Sum of squares	df	Mean square	*F*‐value	*p*	
EE	**Model**	424.74	8	53.09	39.49	< 0.0001	Significant
	Linear mixture	18.65	1	18.65	13.87	0.0039	
	AB	77.87	1	77.87	57.92	< 0.0001	
	AC	10.85	1	10.85	8.07	0.0175	
	BC	74.19	1	74.19	55.18	< 0.0001	
	ABC	6.00	1	6.00	4.46	0.0608	
	AC^2^	231.41	1	231.41	172.12	< 0.0001	
	bc ^2^	9.51	1	9.51	7.08	0.0239	
	ABC^2^	198.40	1	198.40	147.56	< 0.0001	
	**Residual**	13.44	10	1.34			
	Lack of fit	5.37	5	1.07	0.6649	0.6674	Not significant
	Pure error	8.08	5	1.62			
	**Cor total**	438.18	18				
	C.V.: 1.53%, *R* ^2^ = 0.9693, adjusted *R* ^2^ = 0.9448, predicted *R* ^2^ = 0.8975						
EY	**Model**	364.47	8	45.56	186.32	< 0.0001	Significant
	^(1)^ Linear mixture	53.87	1	53.87	220.32	< 0.0001	
	AB	7.14	1	7.14	29.21	0.0003	
	AC	1.62	1	1.62	6.63	0.0277	
	BC	42.54	1	42.54	173.96	< 0.0001	
	ABC	3.19	1	3.19	13.06	0.0047	
	AC^2^	42.35	1	42.35	173.19	< 0.0001	
	BC^2^	52.84	1	52.84	216.11	< 0.0001	
	ABC^2^	42.50	1	42.50	173.82	< 0.0001	
	**Residual**	2.45	10	0.2445			
	Lack of fit	1.37	5	0.2746	1.28	0.3963	Not significant
	Pure error	1.07	5	0.2144			
	**Cor total**	366.92	18				
	C.V.: 0.8117%, *R* ^2^ = 0.9933, adjusted *R* ^2^ = 0.9880, predicted *R* ^2^ = 0.9014						
Antioxidant activity	**Model**	0.4034	8	0.0504	61.04	< 0.0001	Significant
	^(1)^ Linear mixture	0.2756	1	0.2756	333.66	< 0.0001	
	AB	0.0145	1	0.0145	17.60	0.0018	
	AC	0.0104	1	0.0104	12.55	0.0053	
	BC	0.0014	1	0.0014	1.70	0.2215	
	ABC	0.0046	1	0.0046	5.59	0.0397	
	AC^2^	0.0023	1	0.0023	2.76	0.1279	
	BC^2^	0.0007	1	0.0007	0.7939	0.3939	
	ABC^2^	0.0104	1	0.0104	12.62	0.0052	
	**Residual**	0.0083	10	0.0008			
	Lack of fit	0.0013	5	0.0003	0.1848	0.9563	Not significant
	Pure error	0.0070	5	0.0014			
	**Cor total**	0.4117	18				
	C.V.: 0.7320%, *R* ^2^ = 0.9799, adjusted *R* ^2^ = 0.9639, predicted *R* ^2^ = 0.9034						

Depending on the ANOVA test, the three models are appropriate for predicting EE, EY, and antioxidant activity responses since significant *p* values (*p* < 0.0001), non‐significant lack of fit values (*p* > 0.05), the *R* ^2^ values (> 0.96) including adjusted *R* ^2^ (> 0.94) and predicted *R* ^2^ (> 0.89) were all satisfactory (Shahidi Noghabi and Molaveisi 2020). Furthermore, the experimental data were acceptable based on the coefficient of variation (CV) < 10%.

### 3.3 Optimization and Validation Studies

**FIGURE 1:**
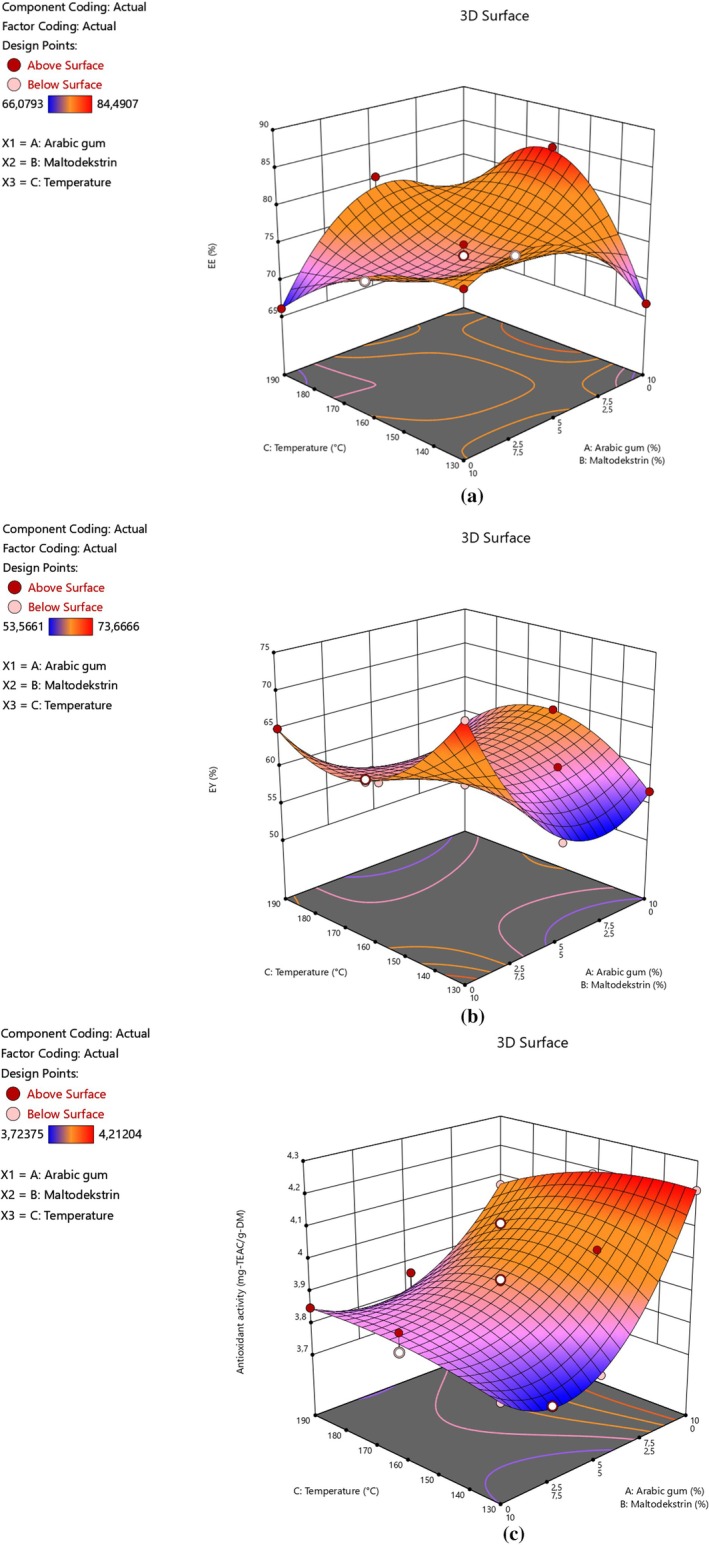
Effects of Arabic gum: Maltodextrin concentration to temperature on the (a) encapsulation efficiency, (b) encapsulation yield, and (c) antioxidant activity of the microcapsules containing polyphenols of rosehip seed extract.

Figure 1 consists of 3D surfaces or contour plots used as the main graphs in mixture designs. Figure 1a shows the effect of mixture components (A as Arabic gum ratio and B as maltodextrin ratio) and process factor (C as inlet temperature) on the EE. The greatest amount of microencapsulation efficiency (84.49%) was observed with Arabic gum alone under mild temperature (160°C). The high EE amounts (> 75%) were observed by using a blend of Arabic gum and maltodextrin under even high heat conditions (190°C). The mixture of Arabic gum and maltodextrin might be causing a powerful matrix to prevent the escape of the polyphenols from the capsules. Similar observations were also reported by Shahidi Noghabi and Molaveisi (2020), Felix et al. (2017), Akhavan Mahdavi et al. (2016), and González‐Peña et al. (2021).

Considering the inlet temperature effect, there was an increase at first as seen in Figure 1a. Later, the yield started to drop. Similarly, Tan et al. reported an increase from 130°C to 140°C (Tan et al. 2015). After that, they also saw a decrease in the yield. They explained this finding with the temperature values of the glass transition and the microparticle. Stickiness occurs when the microparticle's temperature increases more quickly than the glass transition temperature. This situation leads to a decrease in microencapsulation efficiency. Nambiar et al. also reported a comparable impact of inlet temperature on the microencapsulation of tender coconut water (Nambiar et al. 2017).

Figure 1b shows the variation in EY based on the inlet temperature and mixture constituents. The highest yield was examined by maltodextrin alone as wall material. Sarabandi et al. encapsulated eggplant peel extract using identical materials at an inlet temperature of 170°C. They also looked at the effects of comparable mixture components (Sarabandi et al. 2019). They reported that substituting Arabic gum for some or all of the maltodextrin reduced the yield. The samples derived from maltodextrin have a higher yield since it dissolves more easily in solvents with a broad pH range (Kalušević et al. 2017). On the other hand, elevated inlet temperatures lead to an increase in the microencapsulation yield (Figure 1b). Then, the EY was enhanced by temperature. Higher productivity is accessed by elevated drying temperature due to the rapid drying rate. This resulted in enhanced heat and mass transfer (Nambiar et al. 2017). Sticky products with high moisture are formed at mild conditions (León‐Martínez et al. 2010).

In case of the antioxidant activity of the products, the inlet temperature influence does not seem effective as seen in Figure 1c. There is a light increase in the yield. A similar observation on antioxidant activity value measured by the DPPH method was also reported by Nambiar et al. (2017). They also observed that encapsulation efficiency in terms of polyphenols and antioxidant activity reacted differently against temperature. This might be explained by the fact that antioxidant capacity includes several bioactive materials (acids, aromatic compounds, aldehydes, and esters) rather than polyphenols. Another remarkable point in Figure 1c is that antioxidant activity was enhanced sharply by Arabic gum ratio in the biopolymer solution. This might be explained by the own potential antioxidant activity of the Arabic gum contributing to the antioxidant capacity of the microparticles (Ali and EL Said 2020).

The best conditions depending on these considerations were also calculated by Desing‐Expert software to achieve the highest encapsulation efficiency (84.112%), encapsulation yield (63.953%), and DPPH free radical scavenging activity (4.189 mg‐TEAC/g‐DM). Arabic gum alone under 160°C gave the maximum results. In order to verify these conditions, the validation test was held. The difference between the actual and the predicted values was satisfactory (< 2%).

### 3.4 Principal Component Analysis

**FIGURE 2:**
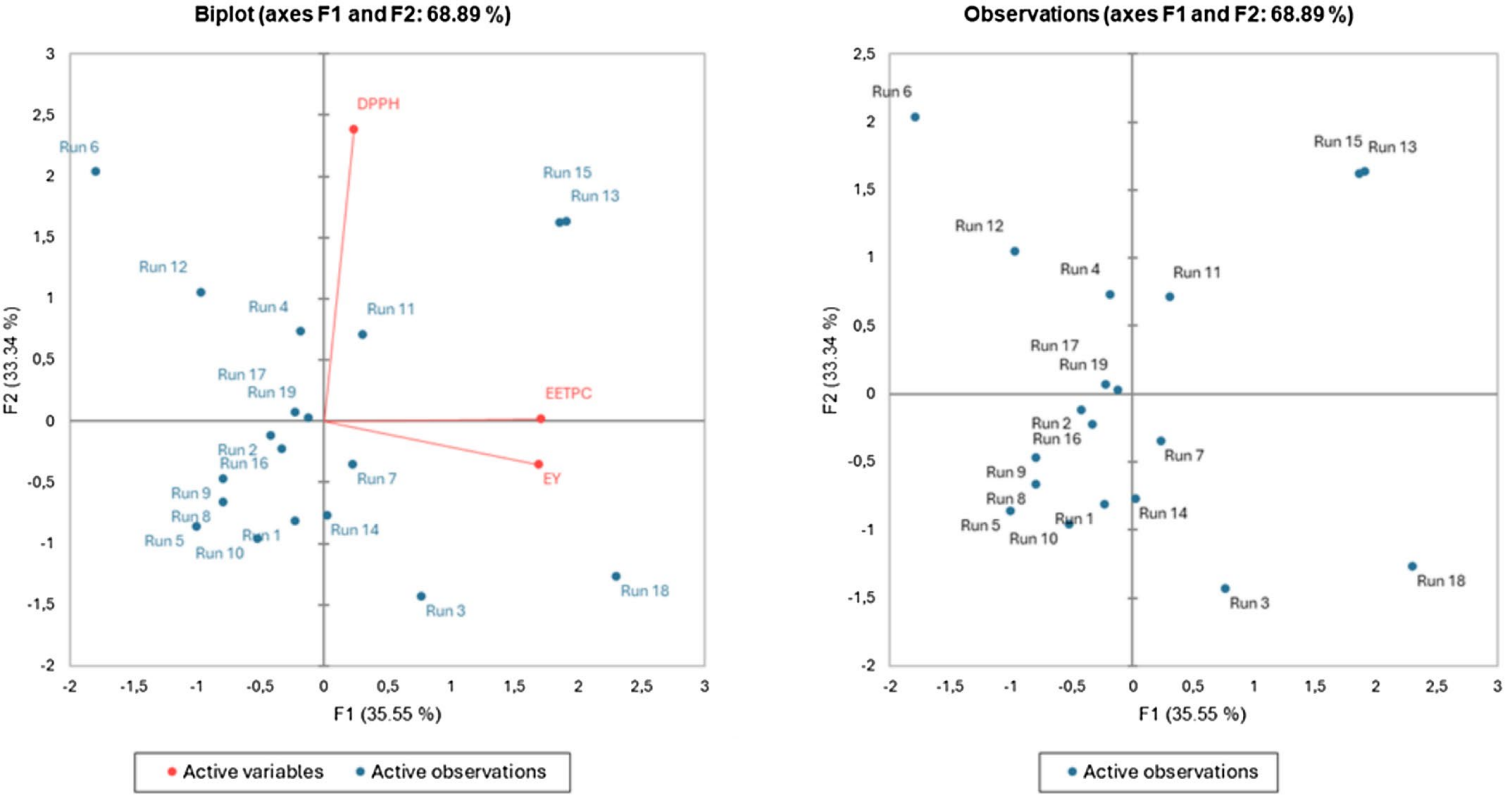
Score plot of principal component analysis for the analysis of encapsulation efficiency, encapsulation yield, and antioxidant activity results of 19 different microencapsules containing polyphenols of rosehip seed extract.

A biplot graph was constructed to visually represent the relationships between variables and observations in the dataset. The biplot graph, as shown in Figure 2, is a graphical display that simultaneously represents both the observations and variables in a multivariate dataset. In this analysis, the first and second components of the biplot explained a substantial portion of the variance in the data. Specifically, the first component accounted for 35.55% of the variance, while the second component explained 33.34% of the variance. These results indicate that a significant proportion of the variability in the dataset can be captured by the relationships between the variables and observations represented in the biplot. The graph illustrates that a high ratio of Arabic gum is associated with enhanced EE and DPPH values, while a high ratio of maltodextrin is linked to increased EY values. EE and DPPH results follow a similar trend, where they are influenced by Arabic gum content when compared to the results of the mixture design. Furthermore, it can be said that EY values are related with temperature and maltodextrin.

### 3.5 Quality of the Products Obtained at Optimum Conditions

Quality analysis of the powder samples obtained under optimum conditions was carried out. For this purpose, powders were analyzed for water activity, moisture content, bulk density, tapped density, and Carr index, solubility, particle size distribution, and morphology. Water activity and moisture content are very important quality parameters for powder samples. They are critical values for followability, stickiness, and agglomeration, as well as determining the shelf life of powder samples obtained by the spray‐drying method. The moisture content and water activity values of rosehip seed extract microcapsules produced under optimum conditions by the spray‐drying method were determined as 4.71% ± 0.05% and 0.24 ± 0.02, respectively. Although both values are highly affected by the spray‐drying process parameters (especially the temperature), the results are consistent with those in the literature (Fournaise et al. 2020; Phisut 2012; Quek et al. 2007).

**FIGURE 3:**
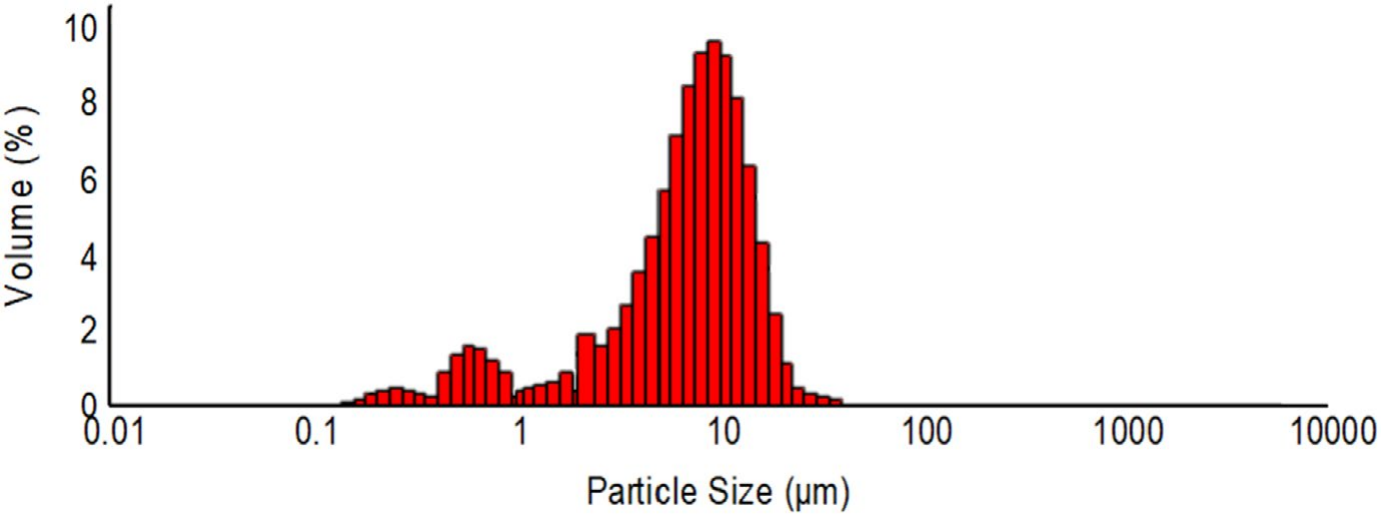
Particle size distribution of the microcapsules containing polyphenols of rosehip seed extract.

The particle size distribution of spray‐dried particles was determined by *d*_10_, *d*_50_, *d*_90_, *D*_[4,3]_ and span values. These values were measured as 1.37 ± 0.15, 7.71 ± 0.01, 14.68 ± 0.08, 8.18 ± 0.04, and 1.73 ± 0.01 μm, respectively. Additionally, the particle size distribution is given by Figure 3. The particle size distributions are unimodal, as seen in Figure 3.

**FIGURE 4:**
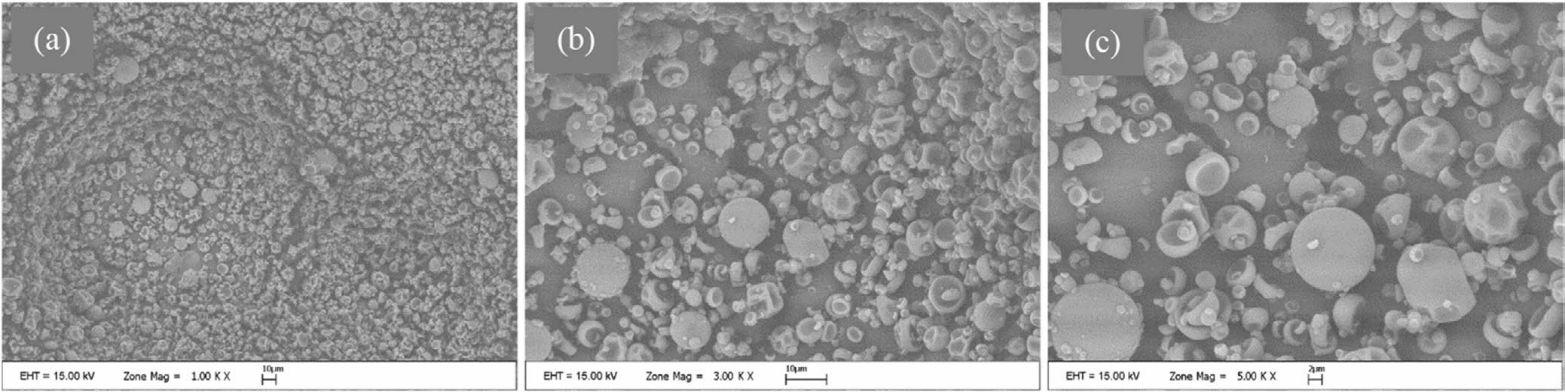
Scanning electron microscopy images of the microcapsules containing polyphenols of rosehip seed extract with different magnifications: (a) 1.00K, (b) 3.00K, (c) 5.00K magnification.

SEM images of the microcapsules are shown in Figure 4. It can be seen that the microcapsules mostly have the same morphological characterization and size, confirming the particle size distribution results. The particle size distribution and also the particle morphology have influenced different technological characterizations such as solubility, bulked‐tapped density, carr index. For example, very fine powders tend to have low wettability, allowing stickiness for a longer time (Etzbach et al. 2020). Solubility is another important quality property of powder product. It especially provides information about the processability of powder products into various products containing the water phase. The solubility values of microcapsules produced under optimum conditions were analyzed as 95.03%. In accordance with several studies (Cano‐Chauca et al. 2005; Fournaise et al. 2020), solubility should be > 90% of powders produced with different carrier material by using spray‐drying.

Bulk and tapped density, and Carr Index of powders are variables that indicate the weight of powder that can fit in a container. They also affect the packaging, storage, transportation, and flowability of powders (Mahdi et al. 2020). The bulk density, tapped density, and Carr Index were measured 275.32 ± 3.29 kg/m^3^, 399.58 ± 6.31 kg/m^3^ and 31.08 ± 1.91, respectively. Although these values are highly affected by the carrier material and inlet temperature used in the spray‐drying process, high bulk and tapped density is generally desired since it reduces the volume of the packaging. Therefore, it reduces transportation and production costs (Arya and Kumar 2022; Goula and Adamopoulos 2005; Samsu and Zahir 2020).

### 3.6 In Vitro Release of Polyphenols From Microparticles

Encapsulated polyphenols under optimum conditions were subjected to in vitro release test by simulating the digestion system as gastric fluid and intestinal fluid. Polyphenol release in intestinal fluid at pH 7.4 was slightly more than in the gastric fluid (pH 1.5) (68.47% vs. 65.44%). This might be a matter of wall material's resistance against the acidity conditions of the medium (Zheng et al. 2011). Similar inclination of release was also reported by Šaponjac et al. during the in vitro release test of polyphenols from microparticles containing beetroot pomace extract (39.03% vs. 27.82%) (Tumbas Šaponjac et al. 2016).

### 3.7 Application Study

After the microparticles containing rosehip seed extract were produced by Arabic gum under 160°C, they were applied as a functional food ingredient in two popular food products. Sunflower oil and corn oil were model foods for the current study. In order to prevent auto‐oxidation of fat‐containing food products, synthetic antioxidants are added to deal with this problem. Otherwise, lipid oxidation causes rancid odor and color in the product, which affects the marketing of the product. Instead of synthetic additives, natural plant extracts have been recommended by various studies (Taghvaei and Jafari 2015). However, the application of direct extract into the product has many drawbacks, while encapsulated materials provide stability during food processing and storage, protecting polyphenols from environmental effects, enhancing desirable food properties, and masking unpleasant aroma (Grgić et al. 2020). Table 5 presents the comparative results of treated and untreated oil samples. Different letters in each column indicate statistically significant differences between groups based on one‐way ANOVA followed by Tukey's post hoc test (*p* < 0.05). The induction time of the sunflower oil was enhanced by around 32%, whereas the corn oil's stability against oxidation was improved by more than 60%. Other quality parameters, such as polyphenol level and antioxidant activity of the products, were also improved significantly. There was an 81% rise in the treated sunflower oil comparing to the pure sunflower oil. Additionally, the functional property of the sunflower oil was improved by ~27% in terms of antioxidant activity. The functional properties (TPC and antioxidant activity) of the corn oil were increased by almost 50%.

**TABLE 5:** Changes in qualitative parameters of sunflower oil and corn oil treated with microparticles.*

Product		IT (h)	TPC (mg‐GAE/g‐OS)	Anitoxidant activity (mg‐TEAC/g‐OS)
Sunflower oil	Untreated	0.69 ± 0.006 a**	0.041 ± 0.001 a	0.041 ± 0.002 a
	Treated	0.91 ± 0.006 b	0.075 ± 0.002 b	0.052 ± 0.002 b
Corn oil	Untreated	0.84 ± 0.010 c	0.036 ± 0.001 a	0.044 ± 0.002 a
	Treated	1.35 ± 0.035 d	0.055 ± 0.004 c	0.064 ± 0.005 c

* Data are given as the arithmetic mean of three replicates.

** Means that do not share a letter in each column are significantly different.

## 4 Conclusion

The findings of the current study pointed that rosehip seed polyphenols were efficiently encapsulated in Arabic gum/maltodextrin (66.08%–84.49% EE, 53.57%–73.67% EY and 3.72–4.21 mg‐TEAC/g‐DM antioxidant activity). The recommended optimum conditions were reliable according to the verification study (the actual − the predicted < 2%). Different formulations were effectively grouped based on their observed variables using chemometric approach. Additionally, SEM findings confirmed the particle size distribution results. 68.47% of the active materials were released in the SIF, while a discharge of 65.44% was detected in the SGF. The bioactivity of the oil samples was significantly enhanced. Most important of all, the stabilities of the sunflower oil and corn oil were boosted by 32% and 60%, respectively. Despite the need for additional research, these results will serve as useful background knowledge before moving on to in vivo and clinical trials.

## Author Contributions

İrem Toprakçı: software, validation, formal analysis, methodology. Ebru Kurtulbaş: formal analysis, validation. Mehmet Torun: validation, formal analysis, methodology. Selin Şahin: conceptualization, methodology, writing – review and editing. Seid Reza Falsafi: project administration, validation, writing – review and editing.

## Funding

The authors have nothing to report.

## Ethics Statement

The authors have nothing to report.

## Conflicts of Interest

The authors declare no conflicts of interest.

## Data Availability

The datasets generated during and/or analyzed during the current study are available from the corresponding author on reasonable request.
